# Personalized tacrolimus therapy in allogeneic hematopoietic stem cell transplantation: from pharmacokinetic variability to novel control strategies

**DOI:** 10.1186/s40780-026-00581-3

**Published:** 2026-05-02

**Authors:** Naoki Yoshikawa

**Affiliations:** Department of Clinical Pharmacy, Oita University Hospital, 1-1 Idaigaoka, Hasama, Yufu, Oita 879-5593 Japan

**Keywords:** Tacrolimus, Allogeneic hematopoietic stem cell transplantation, Graft-versus-host disease, Therapeutic drug monitoring, Pharmacokinetics, FK506-binding protein, Cytochrome P450 3A5, Time in therapeutic range

## Abstract

**Background:**

Allogeneic hematopoietic stem cell transplantation is an established curative therapy for many hematological diseases, but graft-versus-host disease remains a major cause of morbidity and mortality. Tacrolimus, a calcineurin inhibitor, is widely used for prophylaxis because it suppresses T-cell activation. However, its clinical use is complicated by a narrow therapeutic window and marked pharmacokinetic variability. Therapeutic drug monitoring based on trough whole-blood concentrations is routinely used to guide dosing, but this approach has limitations, particularly in transplantation recipients who experience rapid physiological and hematological changes. This review summarizes recent insights into determinants of tacrolimus pharmacology in hematopoietic stem cell transplantation and discusses emerging perspectives for individualized dosing.

**Main body:**

Tacrolimus exerts its immunosuppressive effects by forming a complex with FK506-binding proteins that inhibits calcineurin and suppresses activation of nuclear factor of activated T cells. Beyond this canonical mechanism, interactions with FK506-binding proteins influence the distribution of tacrolimus within blood cells. Because tacrolimus strongly divides into erythrocytes and leukocytes, whole-blood concentrations reflect systemic exposure and drug binding within circulating blood components. In recipients of hematopoietic stem cell transplantation, marked fluctuations in blood cell counts during conditioning therapy and hematopoietic recovery can alter this distribution, potentially causing changes in concentrations without corresponding changes in pharmacologically active exposure. Genetic variation in drug-metabolizing enzymes further contributes to variability in tacrolimus pharmacokinetics. In particular, polymorphisms in the gene encoding cytochrome P450 3A5 influence tacrolimus metabolism and may affect early dose requirements during the post-transplant period. Additionally, temporal fluctuations in tacrolimus exposure within individual patients are increasingly recognized as clinically relevant. Measures that capture the proportion of time during which concentrations remain within the therapeutic range provide a useful indicator of exposure stability.

**Conclusion:**

Tacrolimus therapy after hematopoietic stem cell transplantation is influenced by molecular pharmacology, blood cell-dependent distribution, genetic determinants of metabolism, and temporal variability in drug exposure. Integrating these factors may improve understanding of therapeutic drug monitoring and promote more individualized strategies to maintain stable immunosuppression and improve transplant outcomes.

## Background

Allogeneic hematopoietic stem cell transplantation (HSCT) is an established curative treatment for various hematological malignancies and non-malignant disorders. Despite substantial advances in conditioning regimens, donor selection, and supportive care, graft-versus-host disease (GVHD) is a major cause of transplant-related morbidity and mortality. As acute GVHD is mainly mediated by effector T cells, prevention strategies have focused on suppressing T cells in the recipient [1]. Effective immunosuppressive therapy is essential for successful transplantation outcomes.

Tacrolimus, a calcineurin inhibitor, is widely used as a basis of GVHD prophylaxis. By forming a complex with FK506-binding protein (FKBP) and inhibiting calcineurin phosphatase activity, tacrolimus suppresses T-cell activation and cytokine production, preventing alloreactive immune responses [2, 3]. However, the clinical use of tacrolimus is complicated by a narrow therapeutic window [4, 5]. Inadequate exposure increases the risk of acute GVHD, whereas excessive exposure is associated with nephrotoxicity, neurotoxicity, and opportunistic infections.

To balance efficacy and safety, therapeutic drug monitoring (TDM) of tacrolimus has been routinely implemented, with dose adjustments guided primarily by trough whole-blood concentrations. Although this strategy has improved clinical outcomes compared with fixed dosing, it has limitations. Tacrolimus pharmacokinetics exhibit substantial inter- and intra-patient variability [6], and trough concentrations represent a single time point that may not adequately reflect overall drug exposure. These limitations are particularly pronounced in HSCT recipients. Unlike solid organ transplant patients, HSCT recipients experience profound and dynamic physiological changes, including hematopoietic ablation and reconstitution, systemic inflammation, fluctuating hepatic and renal function, and extensive polypharmacy. Such factors can markedly influence tacrolimus absorption, distribution, metabolism, and elimination over time. Consequently, tacrolimus concentrations often fluctuate widely within individual patients, even under stable dosing conditions.

In recent years, attention has shifted toward a more comprehensive understanding of tacrolimus therapy that extends beyond conventional trough-based TDM. Pharmacogenetic factors, particularly polymorphisms in cytochrome P450 3 A (CYP3A) enzymes, affect tacrolimus metabolism and initial dose requirements [7, 8]. Additionally, the extensive distribution of tacrolimus into blood cells complicates interpretation of whole-blood concentrations, particularly in HSCT recipients with rapidly changing hematological profiles [9–14].

Furthermore, emerging evidence suggests that time-dependent exposure metrics of immunosuppressive drugs, such as intra-patient variability, may be more closely associated with clinical outcomes than isolated trough concentrations [6]. These concepts emphasize the importance of maintaining stable and adequate immunosuppression over time, rather than merely achieving target concentrations at discrete sampling points.

Against this background, there is growing interest in innovative dosing strategies that incorporate systems pharmacology and control theory to achieve personalized tacrolimus therapy. Such approaches aim to proactively control drug exposure in response to dynamic patient-specific changes, representing a paradigm shift from conventional reactive TDM. This review integrates recent clinical and translational evidence to provide an updated framework for personalized tacrolimus management in allogeneic HSCT.

## Main text

### Molecular pharmacology of tacrolimus: Implications beyond systemic exposure

Tacrolimus is a calcineurin inhibitor widely used for immunosuppression in transplantation. Its pharmacological activity is mediated through binding to members of the FKBP family, particularly FKBP12. After entering cells, tacrolimus forms a complex with FKBP12, and this tacrolimus-FKBP complex inhibits the phosphatase activity of calcineurin. As a consequence, dephosphorylation and nuclear translocation of nuclear factor of activated T cells are suppressed, leading to reduced transcription of interleukin-2 and other cytokines essential for T-cell activation and proliferation [2, 3]. This molecular pathway represents the canonical mechanism underlying the immunosuppressive effects of tacrolimus and forms the pharmacological basis for its clinical use in transplantation medicine.

Despite this well-established mechanism, clinical observations indicate that tacrolimus blood concentrations alone do not fully explain variability in immunosuppressive effects. Patients with similar whole-blood concentrations may exhibit markedly different clinical responses, suggesting that additional pharmacodynamic and distributional factors contribute to drug activity. One key determinant is the interaction between tacrolimus and FKBP proteins, which function as intracellular binding partners and as modulators of tacrolimus distribution within blood components. Experimental studies have demonstrated that tacrolimus exhibits strong binding to erythrocytes, and this binding is influenced by FKBP proteins present within blood cells [9, 10, 15]. In particular, FKBP12 expressed in erythrocytes contributes to intracellular sequestration of tacrolimus, influencing the relationship between plasma exposure and measured whole-blood concentrations. Investigations using human erythrocytes have revealed that inhibition or saturation of intracellular FKBP alters tacrolimus distribution between plasma and cellular compartments, indicating that FKBP-mediated binding plays a central role in the drug’s blood partitioning characteristics [16].

In addition to intracellular FKBP, extracellular FKBP may influence tacrolimus pharmacology. Previous studies suggested that extracellular FKBP can interact with tacrolimus before cellular uptake, potentially affecting drug distribution and binding dynamics within circulating blood cells [16]. These interactions may contribute to variability in tacrolimus measurements obtained through whole-blood therapeutic drug monitoring. Furthermore, variability may occur independent of actual changes in pharmacologically active exposure at the cellular target level, highlighting a critical implication for tacrolimus pharmacology: measured whole-blood concentrations reflect systemic drug exposure and complex binding interactions with FKBP proteins in circulating blood cells.

From a clinical perspective, this molecular framework provides a comprehensive understanding of why tacrolimus therapeutic drug monitoring sometimes fails to fully predict clinical outcomes. Variability in FKBP-mediated binding may partially explain discrepancies between concentration measurements and immunosuppressive effects observed in transplant recipients. Recognizing the role of FKBP in tacrolimus distribution expands the traditional pharmacokinetic interpretation of tacrolimus concentrations and underscores the importance of integrating molecular pharmacology into individualized immunosuppressive therapy.

### Blood cell-dependent distribution and interpretation of whole-blood concentrations

Tacrolimus exhibits distinctive pharmacokinetic characteristics that complicate the interpretation of TDM results. One of the most important features is its extensive distribution into blood cells, particularly erythrocytes [12, 17] and leukocytes, resulting in high blood-to-plasma ratio [18]. Because tacrolimus TDM is almost universally based on whole-blood measurements, understanding the determinants of blood cell-dependent distribution is essential for accurate interpretation of concentration data. The binding of tacrolimus to blood cells is influenced by several factors, such as hematocrit [10–14]. In stable patient populations, these factors remain relatively constant, allowing whole-blood concentrations to considerably surrogate for systemic exposure. Contrastingly, allogeneic HSCT recipients experience profound and dynamic changes in blood cell composition, particularly during the peri-transplant period.

Conditioning regimens induce severe cytopenia, followed by gradual hematopoietic recovery and immune reconstitution. During this process, hematocrit and leukocyte counts can change markedly over short time intervals. Our retrospective analyses demonstrated significant correlations between tacrolimus whole-blood concentrations and variations in blood cell counts, independent of dose adjustments [19], suggesting that apparent fluctuations in measured tacrolimus concentrations may be driven, at least in part, by redistribution between plasma and cellular compartments rather than true changes in pharmacologically active exposure. This phenomenon has important clinical implications. Increases in whole-blood tacrolimus concentrations accompanying hematopoietic recovery may prompt clinicians to reduce the dose, potentially leading to under-immunosuppression and increased risk of GVHD. Conversely, reducing whole-blood tacrolimus concentrations during cytopenic phases may lead to aggressive dose escalation, increasing the risk of tacrolimus-related toxicities once blood cell counts recover. Such misinterpretation is particularly problematic during the early post-transplant period, when hematological instability and GVHD risk are highest.

These observations highlight a critical limitation of conventional tacrolimus TDM in HSCT. Whole-blood concentrations should not be interpreted in isolation but in conjunction with contemporaneous hematological data. Awareness of blood cell-dependent distribution may improve clinical decision-making and reduce unnecessary dose adjustments, underscoring the potential value of alternative exposure metrics or model-based approaches that account for dynamic changes in blood cell composition. In summary, blood cell-dependent distribution is a key source of apparent pharmacokinetic variability in tacrolimus therapy among HSCT recipients. Recognizing and accounting for this occurrence is essential for accurate interpretation of TDM data and is a basis for more sophisticated and individualized dosing strategies.

### Pharmacogenetic determinants of tacrolimus pharmacokinetics in HSCT

Tacrolimus pharmacokinetics are characterized by marked inter-individual variability, and genetic polymorphisms in drug-metabolizing enzymes is one of the most important intrinsic determinants of this variability [7, 8]. Tacrolimus is primarily metabolized by CYP3A enzymes, particularly CYP3A4 and CYP3A5, and is a substrate of the efflux transporter P-glycoprotein (ABCB1) [20]. Among these factors, polymorphisms in the *CYP3A5* gene have been most consistently associated with differences in tacrolimus clearance. The CYP3A5*3 allele results in aberrant mRNA splicing and the absence of functional CYP3A5 protein [21, 22]. Individuals carrying this allele (non-expressers) exhibit reduced tacrolimus metabolism and consequently higher dose-normalized blood concentrations compared with those expressing CYP3A5. In solid organ transplantation, the clinical relevance of this polymorphism is well established, and multiple studies have demonstrated that CYP3A5 expressers require substantially high tacrolimus doses to achieve target concentrations. Genotype-guided initial dosing has therefore been proposed and implemented in some clinical settings to accelerate attainment of therapeutic exposure. In allogeneic HSCT, however, the influence of pharmacogenetic factors on tacrolimus pharmacokinetics is more complex and less predictable. The frequent use of concomitant medications, particularly azole antifungal agents, further complicates tacrolimus metabolism through potent inhibition of CYP3A enzymes [23–25].

Despite these confounding factors, emerging evidence indicates that *CYP3A5* polymorphism is a clinically relevant determinant of tacrolimus exposure in HSCT, particularly during the early post-transplant period [26]. During this phase, tacrolimus pharmacokinetics are highly unstable, and rapid optimization of immunosuppressive exposure is critical for effective GVHD prophylaxis. Our clinical analyses demonstrated that the CYP3A5*3 polymorphism significantly influenced the concentration-dose relationship of tacrolimus and was associated with GVHD outcomes when early underexposure was not adequately corrected [26], suggesting that the clinical utility of pharmacogenetic information in HSCT lies primarily in guiding initial dosing strategies before sufficient therapeutic drug monitoring data become available. Incorporation of *CYP3A5* genotype into early tacrolimus dose selection may facilitate faster achievement of therapeutic exposure, reduce the duration of underexposure, and improve the continuity of immunosuppression during a critical therapeutic opportunity. Although pharmacogenetics alone cannot fully account for the complex pharmacokinetics of tacrolimus in HSCT, it is an important component of a multifactorial approach to personalized immunosuppressive therapy.

### Intra-patient variability and the concept of time in therapeutic range

In addition to inter-individual differences, intra-patient variability (IPV) in tacrolimus exposure is a clinically important determinant of outcomes in transplantation medicine. IPV reflects temporal fluctuations in drug concentrations within the same individual and is influenced by multiple factors, including physiological changes, drug-drug interactions, adherence, and variability in absorption and metabolism [27–29]. In allogeneic HSCT, IPV is particularly pronounced because patients undergo rapid and substantial changes in clinical status during the peri-transplant period. High IPV has been associated with adverse outcomes in solid organ transplantation, such as rejection and graft loss, suggesting that unstable immunosuppressive exposure compromises consistent immune control [5, 30, 31]. In HSCT, similar concerns exist, as fluctuations in tacrolimus exposure may result in periods of under-immunosuppression that predispose patients to GVHD, or over-immunosuppression that increases the risk of toxicity and infection.

Traditional metrics used to quantify IPV, such as the coefficient of variation or standard deviation of trough concentrations, describe the degree of dispersion but do not indicate whether concentrations remain within the therapeutic range. As a result, these metrics provide limited insight into the clinical adequacy of tacrolimus exposure. To address this limitation, the concept of time in therapeutic range (TTR) has been introduced as a more clinically intuitive measure of exposure stability. TTR is the proportion of time during which drug concentrations remain within a predefined therapeutic window. By integrating the magnitude and duration of exposure, TTR captures the continuity of immunosuppressive coverage rather than isolated concentration values. Previous studies have reported on the association between the TTR of tacrolimus blood concentration and the efficacy of tacrolimus in inhibiting rejection post-organ transplantation [32–37]. In HSCT recipients, early post-transplant exposure is particularly critical, as this period is characterized by intense alloimmune activation and high GVHD risk. Our clinical studies demonstrated that higher TTR during the early post-transplant period was significantly associated with a reduced incidence and severity of acute GVHD [38], suggesting that maintaining tacrolimus concentrations within the therapeutic range over time is more clinically relevant than achieving target trough concentrations at sporadic sampling points.

The TTR concept provides a practical framework for evaluating and optimizing tacrolimus dosing strategies. Unlike single-point measurements, TTR aligns closely with clinical objectives and is a meaningful endpoint in observational and interventional studies. Furthermore, TTR naturally integrates with emerging model-informed and control-based dosing approaches aimed at stabilizing exposure in dynamic clinical settings. In summary, IPV represents a key challenge in tacrolimus therapy for HSCT recipients, and TTR offers a robust and clinically relevant metric to address this challenge. Emphasizing time-dependent exposure control may facilitate more consistent immunosuppression and contribute to improved GVHD prophylaxis and overall transplant outcomes.

## Conclusions

Tacrolimus is the basis of GVHD prophylaxis in allogeneic HSCT, yet its clinical management is challenged by substantial pharmacokinetic variability due to multiple interacting determinants, including molecular pharmacology, pharmacogenetic factors, dynamic hematological changes, and intra-patient fluctuations in drug exposure. Collectively, these factors complicate the interpretation of whole-blood tacrolimus concentrations and limit the effectiveness of conventional therapeutic drug monitoring strategies. Emerging evidence indicates that tacrolimus exposure should be understood within a broader and more dynamic framework. Pharmacogenetic determinants, particularly *CYP3A5* polymorphism, contribute to variability in early drug metabolism, while shifts in blood cell counts can influence the distribution of tacrolimus and the interpretation of whole-blood measurements. Moreover, the concept of intra-patient variability highlights the importance of maintaining stable immunosuppressive exposure over time. Metrics such as TTR provide a more clinically meaningful representation of exposure continuity and have been associated with improved GVHD outcomes. Collectively, these insights support a transition from reactive dose adjustment toward proactive and adaptive dosing strategies. Approaches informed by pharmacogenetics and physiological interpretation of concentration data may enable more consistent tacrolimus therapy. Such integrated strategies enhance GVHD prophylaxis and contribute to improved outcomes in HSCT recipients.

## Data Availability

No datasets were generated or analysed during the current study.

## Abbreviations

HSCT: Hematopoietic stem cell transplantation
GVHD: Graft-versus-host disease
TTR: Time in therapeutic range
IPV: Intra-patient variability
TDM: Therapeutic drug monitoring
CYP3A: Cytochrome P450 3 A
